# Impact of reductive tricuspid ring annuloplasty on right ventricular size, geometry and strain in an ovine model of functional tricuspid regurgitation

**DOI:** 10.1093/icvts/ivac187

**Published:** 2022-07-04

**Authors:** Artur Iwasieczko, Marcin Malinowski, Monica Solarewicz, Jared Bush, Brian MacDougall, Manuel Rausch, Tomasz A Timek

**Affiliations:** Division of Cardiothoracic Surgery, Spectrum Health, Michigan State College of Human Medicine, Grand Rapids, MI, USA; Clinical Department of Cardiac Surgery, District Hospital No. 2, University of Rzeszow, Rzeszow, Poland; Department of Cardiac Surgery, Medical University of Silesia, School of Medicine in Katowice, Katowice, Poland; Division of Cardiothoracic Surgery, Spectrum Health, Michigan State College of Human Medicine, Grand Rapids, MI, USA; Division of Cardiothoracic Surgery, Spectrum Health, Michigan State College of Human Medicine, Grand Rapids, MI, USA; Division of Cardiothoracic Surgery, Spectrum Health, Michigan State College of Human Medicine, Grand Rapids, MI, USA; Department of Aerospace Engineering & Engineering Mechanics, Department of Biomedical Engineering, Institute of Computational Engineering and Science, University of Texas at Austin, Austin, TX, USA; Division of Cardiothoracic Surgery, Spectrum Health, Michigan State College of Human Medicine, Grand Rapids, MI, USA

**Keywords:** Tricuspid valve, Tricuspid ring annuloplasty, Right ventricle, Geometry, Strains

## Abstract

**OBJECTIVES:**

Reductive ring annuloplasty of the tricuspid annulus represents the contemporary surgical approach to functional tricuspid regurgitation (FTR). We set out to investigate the influence of moderate reductive tricuspid ring annuloplasty on tricuspid regurgitation and right ventricular (RV) size, geometry and strain in an ovine model of chronic FTR.

**METHODS:**

Eight healthy Dorsett male sheep (62.8 + 2kg) underwent a left thoracotomy for placement and tightening of pulmonary artery band to at least double proximal pulmonary artery blood pressure. After 8 weeks of recovery, animals underwent sternotomy, epicardial echocardiography and sonomicrometry crystal implantation. Six crystals were placed around tricuspid annulus and 13 on RV free wall epicardium along 3 parallels defining 3 wall regions (basal, mid and lower) and 1 on the RV apex. All animals underwent beating heart implantation of 26 mm MC3 annuloplasty ring during a second cardiopulmonary bypass run after baseline data acquisition. Simultaneous haemodynamic, sonomicrometry and echocardiography data were acquired at Baseline and after reductive tricuspid ring annuloplasty.

**RESULTS:**

Implantation of reductive ring annuloplasty resulted in 47 ± 7% annular area reduction (996 ± 152 mm vs 516 ± 52 mm^2^, *P* = 0.0002) and significantly decreased RV end-diastolic volume (185 ± 27 vs 165 ± 30 ml, *P* = 0.02). Tricuspid ring annuloplasty effectively reduced FTR grade (3.75 ± 0.6 vs 0.3 ± 0.5, *P* = 0.00004) and had little influence on RV function, cross-sectional area, radius of curvature or free wall regional strains.

**CONCLUSIONS:**

In adult sheep with 8 weeks of pulmonary artery banding and FTR, tricuspid annulus reduction of 47% with prosthetic ring annuloplasty effectively abolished FTR while maintaining regional RV function and strain patterns.

**Figure:**
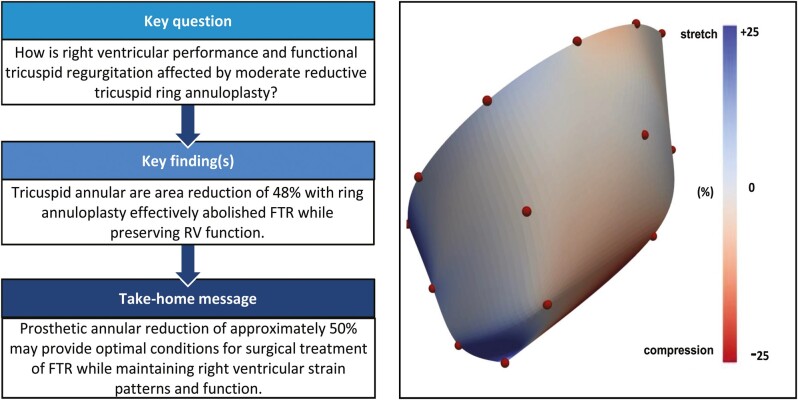

## INTRODUCTION

Functional tricuspid regurgitation (FTR) is commonly observed in patients with left-sided valve disease [1], and until recently, it was believed that correction of left valvular pathology would lead to resolution of tricuspid insufficiency. However, this strategy has been demonstrated to be unreliable in halting progression of tricuspid regurgitation (TR), leaving many patients with untreated significant TR [2]. Uncorrected moderate or severe TR during left heart surgery is associated with increased morbidity and mortality and is a marker of worse long-term survival regardless of left ventricular (LV) ejection fraction or pulmonary artery pressure [3]. Indeed, current cardiovascular guidelines emphasize the need for more aggressive treatment of TR [4] and the increasing incidence of TR in the community has recently been recognized as a ‘public health crisis’ [5]. FTR is primarily associated with tricuspid annular dilatation and right ventricular (RV) enlargement and dysfunction, and contemporary repair of FTR is centred on annular reduction and remodelling with prosthetic rings. However, aggressive annular reduction, as demonstrated in ovine experimental mitral annular reduction, may deleteriously affect regional myocardial performance [6]. This effect may be more pronounced on the more compliant RV myocardium. Indeed, we have recently demonstrated that progressive suture reduction of the tricuspid annulus beyond 50% of baseline area in both normal sheep [7] and those with functional FTR and biventricular failure [8], perturbed regional RV function and strain. In the current study, we set out to investigate the influence of moderate reductive tricuspid ring annuloplasty (TRA) on TR and RV size, geometry and strain in an ovine model of chronic FTR.

## METHODS

### Ethics statement

All animals received humane care in compliance with the Principles of Laboratory Animal Care formulated by the National Society for Medical Research and the Guide for Care and Use of Laboratory Animals prepared by the National Academy of Science and published by the National Institutes of Health. The study was approved by the West Michigan Regional Lab Institutional Animal Care and Use Committee. The approval number for the study was 2020-035.

### Operative procedure

#### Pulmonary artery banding

Eight healthy Dorsett castrated male sheep (62 ± 2) had external left jugular intravenous catheters placed under local anaesthesia using 1% lidocaine. Animals were then anaesthetized with propofol (2–5 mg/kg intravenously), intubated and mechanically ventilated. General anaesthesia was maintained with inhalational isoflurane (1–2.5%). Fentanyl (5–20 mg/kg/min) was infused as additional maintenance of anaesthesia. Using sterile technique, the heart was exposed via limited 10 cm left thoracotomy between 4 and 5 intercostal space. Arterial 18G catheter was introduced to left internal thoracic artery for continuous arterial blood pressure measurement. Baseline epicardial echocardiography was performed to assess biventricular function and tricuspid valve competence. An umbilical tape band was placed around the distal main pulmonary trunk and progressively tightened with clips just until brink of continued haemodynamic stability as previously described [9] to at least double baseline pulmonary artery blood pressure. Proximal pulmonary artery blood pressure was continuously monitored during the procedure with a 20G intravenous catheter placed in the artery. The surgical incision was approximated in standard fashion with 2-0 suture on muscle and subcutaneous tissue, and 4-0 suture to close skin. Intercostal nerves in the region were infiltrated with 0.25% bupivacaine. The animals were taken back from the recovery room to the pen when breathing spontaneously, standing up and eating. Prophylactic antibiotics (cefazolin 2 g intravenously every 12 h and gentamicin 240 mg intravenously every 24 h) were given for 10 days postoperatively beginning with the preoperative dose. The animals were followed for 8 weeks with routine clinical care including medical treatment of right heart failure as needed.

#### Tricuspid ring annuloplasty

**Figure 1:**
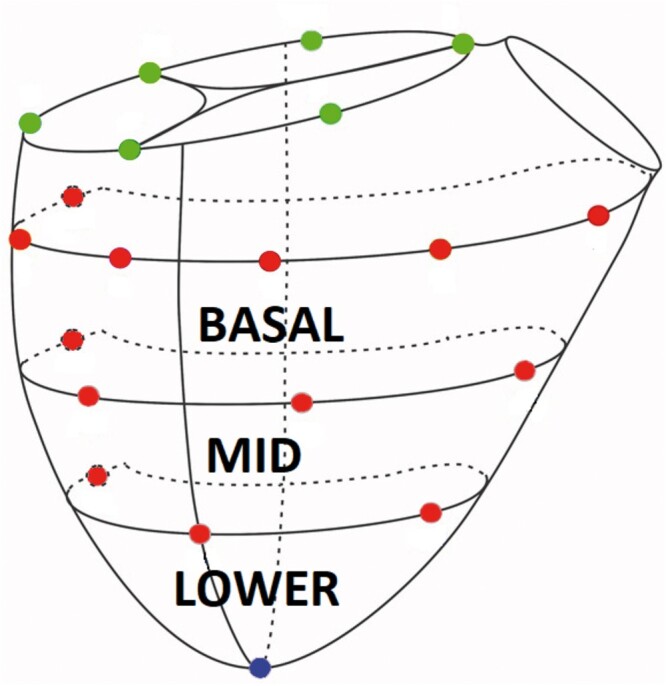
Schematic representation of right ventricular epicardial sonomicrometry crystal array. Green sphere = tricuspid annular crystals, red spheres = right ventricular free wall epicardial crystals and blue sphere = right ventricular apex (A color version of this figure appears in the online version of this article).

**Figure 2:**
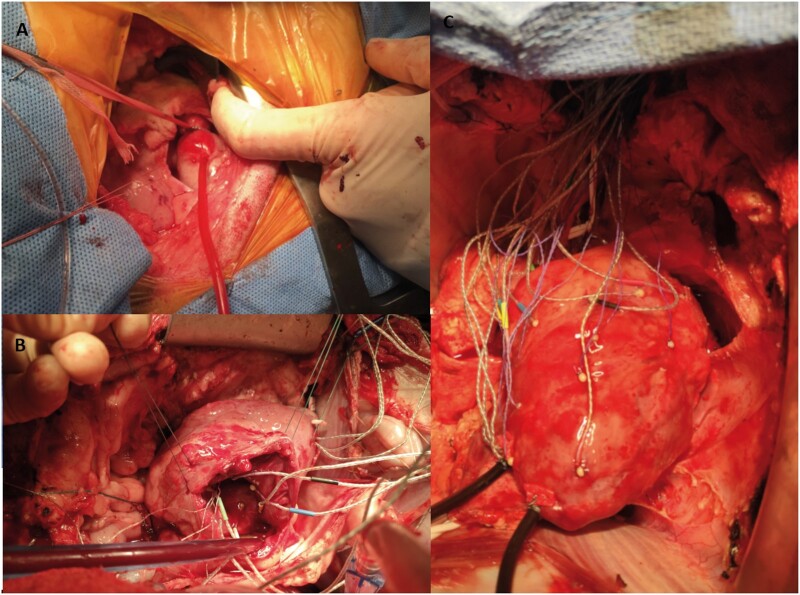
Intraoperative photographs of (**A**) pulmonary artery banding through a left thoracotomy, (**B**) implanted crystals around the tricuspid annulus and (**C**) sonomicrometry crystals on right ventricular epicardium with externalized right ventricular and left ventricular pressure transducers.

After 8 weeks, the sheep were brought back to the operating room and anaesthesia was induced as described above. Using clean technique, the heart was exposed via median sternotomy and baseline epicardial echocardiographic examination was performed to assess biventricular volume, function and FTR. The animal was then fully heparinized (300 µg/kg intravenous bolus) and the right carotid artery and right internal jugular vein were cannulated for cardiopulmonary bypass (CPB). Arterial 18G catheter was introduced to left internal thoracic artery for continuous arterial blood pressure measurement. The heart-lung machine was primed with Plasma-Lyte^®^ or similar crystalloid solution prior to CPB. After activated clotting time exceeded 480 s, CPB was initiated. The procedure was conducted on the beating heart. After snaring both cava, the right atrium was opened and sonomicrometry crystals (2 mm; Sonometrics Inc., London, ON, Canada) were implanted with 5-0 polypropylene suture. Six crystals were placed around the tricuspid annulus. Thirteen crystals were placed on the on the RV free wall along 3 parallels defining 3 wall regions (Basal, Mid and Lower) and 1 at the RV apex as shown schematically in Fig. 1. An electrocardiogram (ECG) electrode was sutured to the LV free wall. Pressure sensors (PA4.5-X6; Konigsberg Instruments, Pasadena, CA, USA) were placed in the RV and LV cavities through the apex and in the right atrium through the atriotomy. Intraoperative images of the procedure are demonstrated in Fig. 2. After closure of the right atrium and when satisfactory haemodynamics were achieved, the animal was weaned from CPB. All data were collected with the chest open. An epicardial echocardiographic examination was repeated, and haemodynamic and sonomicrometric data were collected simultaneously (Baseline). CPB was then reinitiated and reductive TRA was performed through the previously made atriotomy on the beating heart. Reductive annuloplasty was performed using the MC3 26 mm prosthesis (Edwards Lifesciences Corp., Irvine, CA, USA) and interrupted 2-0 ethibond sutures with range of 8–10. The animals were again weaned from CBP, and after achieving haemodynamic stability, simultaneous echocardiographic, haemodynamic, and sonomicrometry data were collected (TRA). At the conclusion of the experiment, the animals were euthanized by exsanguination and infusion of high potassium solution. The heart was excised immediately after exsanguination and anatomical position of all implanted crystals was confirmed.

### Sonomicrometry data acquisition and analysis

Sonomicrometry data were acquired using a Sonometrics Digital Ultrasonic Measurement System DS3 (Sonometrics Corporation, London, ON, Canada) as previously described [10]. Data from 4 consecutive cardiac cycles during normal sinus rhythm were averaged for each animal. Data were acquired at 128 Hz with simultaneous LV pressure, RV pressure, central venous pressure and ECG recordings. All sonomicrometry recordings were analysed offline using CardioSOFT Software, ver. 3.4.82 (Sonometrics Corporation) and custom designed code for MATLAB (MathWorks, Natick, MA, USA). All parameters were calculated at end-systole and end-diastole. End-diastole was defined as the peak of the R-wave on the ECG and end-systole as time of maximum negative dP/dt of LV pressure. RV free wall regional (basal and mid) circumferential, longitudinal and areal strains, and regional (basal, mid and lower) radius of curvature and cross-sectional area were calculated from 3D crystal coordinates as was tricuspid annulus area. RV volume was calculated using convex hull method based on annular and epicardial crystal coordinates. To calculate RV epicardial strains, we used previously described method [11] based on triangularly connected RV free wall epicardial strain mesh derived from crystal coordinates which were recreated using a modified Loop subdivision algorithm [12]. Circumferential, longitudinal and areal strains were calculated according to the methodology described in our recent work [7]. RV free wall cardiac strain was calculated for both Baseline and TRA during the cardiac cycle versus the reference state at end-diastole. RV free wall interventional strain was calculated as the strain at end-diastole and end-systole with TRA versus Baseline at the same time point.

### Echocardiographic data acquisition

Epicardial echocardiography was used to assess RV function and size and TR. Images were acquired with a 1.5- to 4.5-MHz transducer and Vivid S6 ultrasound machine (GE Healthcare, Chicago, IL, USA). After image acquisition, the degree of valvular insufficiency was assessed using American Society of Echocardiography criteria. The grading included comprehensive evaluation of colour flow and continuous-wave Doppler. Tricuspid regurgitation was graded accordingly and categorized by an experienced Echocardiographer as none or trace (0), mild (1), moderate (2), moderate to severe (3) and severe (4).

### Statistical methods

All data are presented as mean ± standard deviation. Comparisons of parameters between Baseline and TRA were performed using Student’s *t*-test for dependent observations with a *P*-value of <0.05 considered significant. Normality was assessed by using Shapiro–Wilk test, normal distribution was confirmed, additionally, Wilcoxon test was implied for repeated measures to confirm data, with *P*-value of <0.05 considered as significant. Data analysis was performed using Statistical Package of Social Science (SPSS) version 26.0 for MacOS.

## RESULTS

### Pulmonary artery banding

Placement of the pulmonary band was associated with acute increase in systolic pulmonary artery pressure from 19.2 ± 7.5 to 57.4 ± 23.1 mmHg (*P* = 0.0001) while mean pulmonary artery pressure increased from 13.1 ± 5.2 to 33.8 ± 13.5 mmHg (*P* = 0.00001). After 8 weeks of pulmonary banding, systolic pulmonary artery pressure decreased to 41.9 ± 6.4 mmHg while the mean pressure decreased to 27.5 ± 3.1 mmHg indicative of progressive RV failure. Echocardiographic parameters acquired before application of the pulmonary artery band and after 8 weeks of banding are summarized in Table 1. Eight weeks of pulmonary banding was associated with significant TR, reduced RV function, increased wall thickness and remarkable tricuspid annulus area and left atrial dilation.

**Table 1:** Pulmonary artery banding

*n* = 8	Pre-PB	Post-PB (8 weeks)	*P*-value
TR (+0–4)	0.56 ± 0.53	3.75 ± 0.71	0.000002
RVFAC (%)	46 ± 20	38 ± 7	0.006
TAPSE	1.06 ± 0.44	0.74 ± 0.13	0.002
RVFW thickness (mm)	0.39 ± 0.15	0.56 ± 0.07	0.0003
TA (cm)	2.1 ± 0.8	3.1 ± 0.2	0.0000008
RAA (mm^2^)	6.8 ± 2.9	14 ± 4.2	0.006
RA Vol (ml)	13.9 ± 6.8	42.3 ± 19.7	0.008

Mean ± SD.

PB: pulmonary artery banding; RA Vol: right atrial volume; RAA: right atrial area; RVFWd: right ventricular free wall; TA: tricuspid annulus; TAPSE: tricuspid annular plane systolic excursion; TR: tricuspid regurgitation: RVFAC: right ventricular fractional change.

### Tricuspid ring annuloplasty

Echocardiographic and haemodynamic data at Baseline following 8 weeks of pulmonary artery banding and after reductive TRA are illustrated in Table 2. TRA effectively abolished FTR without significantly affecting haemodynamics. Our echocardiographic assessment of RV function after implantation of TRA did not suggest deterioration of myocardial performance due to procedural time or prosthesis implantation. Sonomicrometry-derived tricuspid annulus area and RV geometry and size are summarized in Table 3. Implantation of the MC3 tricuspid ring resulted in significant reduction of annular area and RV volume potentially due to obliteration of tricuspid insufficiency. RV geometry, as assessed by cross-sectional area and radius of curvature was unchanged after TRA.

**Table 2.** Haemodynamics

*n* = 8	Baseline	TRA	*P*-value
TR (0–4)	3.75 ± 0.6	0.3 ± 0.5	0.00004
HR (b/min)	106 ± 20	96 ± 22	0.3
LVP (mmHg)	87 ± 17	95 ± 16	0.1
RVP (mmHg)	42 ± 14	43 ± 10	0.5
CVP (mmHg)	12 ± 1	12 ± 2	0.9

Mean ± SD.

CVP: central venous pressure; HR: heart rate; LVP: left ventricular pressure; RVP: right ventricular pressure; TR: tricuspid regurgitation.

**Table 3:** Tricuspid annular and right ventricular geometry

*n* = 8		Baseline	TRA	*P*-value
TAA area (mm^2^)		996 ± 152	516 ± 52	0.0002
TA reduction (%)			47.3 ± 6.9	
RV EDV (ml)		185 ± 27	165 ± 30	0.02
RV CSA (mm^2^)	Basal	4922 ± 567	4853 ± 447	0.7
	Mid	4696 ± 913	4634 ± 750	0.7
	Lower	2158 ± 1208	2286 ± 972	0.5
RV ROC (mm)	Basal	51.4 ± 2.6	51.8 ± 3.5	0.7
	Mid	47 ± 3.7	46.6 ± 5.7	0.9
	Lower	36.6 ± 4.2	37.7 ± 7.6	0.2

Mean ± SD.

CSA: cross-sectional area; EDV: end-diastolic volume; ROC: radius of curvature; RV: right ventricular; TA: tricuspid annulus; TAA: tricuspid annulus area.

### Right ventricular strain

**Figure 3:**
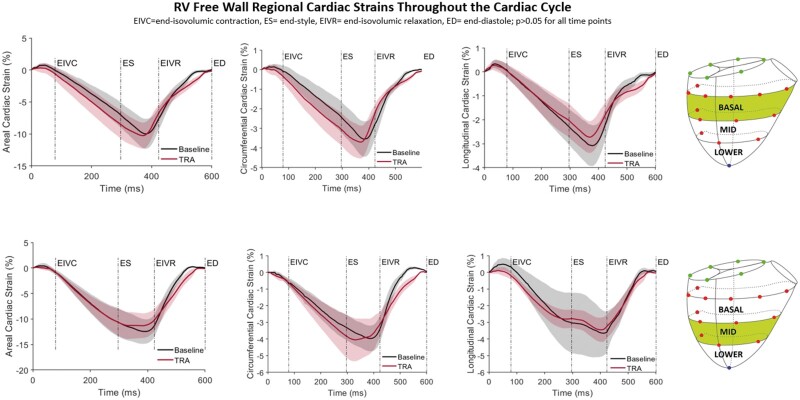
Right ventricular free wall regional epicardial strains. Areal, circumferential and longitudinal cardiac strains for Baseline (black) and tricuspid ring annuloplasty (TRA, red) throughout the cardiac cycle with the reference state at end-diastole. Shadowed areas represent confidence intervals. ED: end-diastole; EIVC: end-isovolumic contraction; ES: end-systole; EIVR: end-isovolumic relaxation (A color version of this figure appears in the online version of this article).

**Figure 4:**
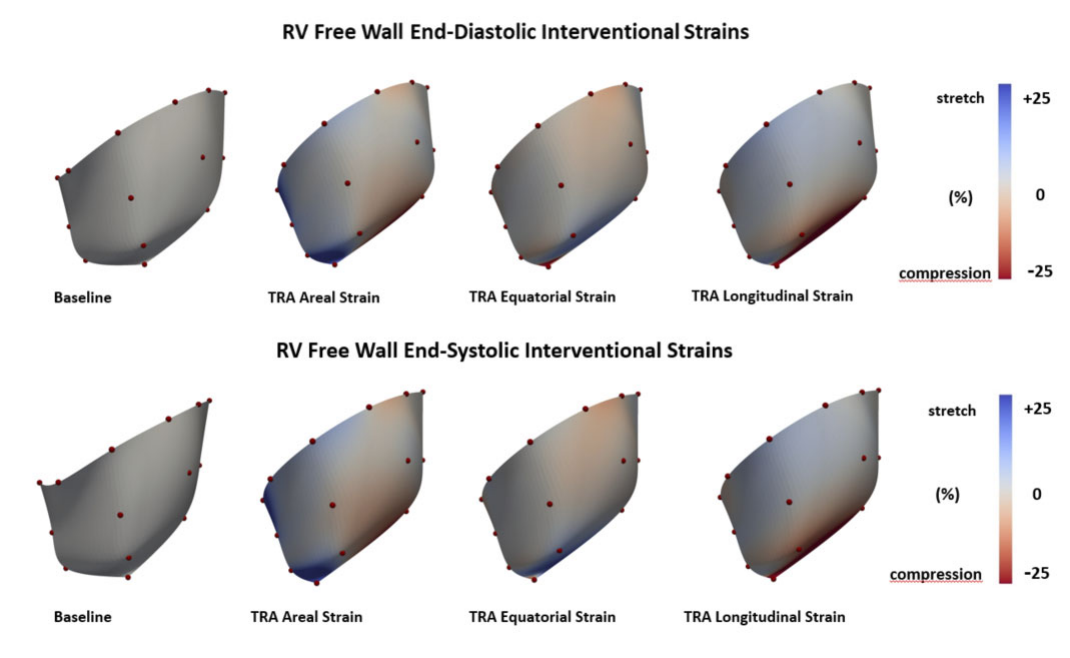
Coloured maps of right ventricular free wall interventional strains. Equatorial, longitudinal and areal epicardial deformation presented at point of end-diastole and end-systole referenced to Baseline. Red colour indicates myocardial compression and blue colour myocardial stretch. Colour tone bars indicate range of deformation (A color version of this figure appears in the online version of this article).

RV free wall circumferential, longitudinal and areal strain in the basal and mid RV regions throughout the cardiac cycle for Baseline and after TRA is presented in Fig. 3. No change in strain patterns throughout the cardiac cycle was observed with TRA. Similarly, cardiac interventional strain at end-diastole and end-systole did not change significantly with TRA as illustrated in Fig. 4.

## DISCUSSION

The current study revealed that moderate tricuspid annular reduction of 47% effectively treated experimental FTR without affecting RV shape, size or regional strain. We have previously demonstrated in healthy ovine hearts [7] that severe annular size reduction with progressive suture annuloplasty beyond 50% of initial size was associated with reduced RV function and regional strains. These initial findings were confirmed in an ovine rapid pacing model of biventricular failure and FTR [8] with annular area reduction of ∼50% found optimal for control of tricuspid insufficiency and maintenance of RV function. The current data corroborate these prior studies in an isolated model of FTR and RV dysfunction using a clinically utilized annular prosthetic ring. These data may be of clinical pertinence as the concept of annular undersizing for repair of functional regurgitation first introduced by Bolling *et al.* [13] continues to evolve. Undersized annular reduction for repair of functional mitral regurgitation has been shown to improve subvalvular geometry but may negatively influence regional LV myocardial performance [14]. Annular undersizing with a prosthetic ring has now been advocated for repair of FTR but aggressive annular restriction may have a greater effect on the right heart due to the more compliant nature of the RV myocardium. Clinically, use of 26 or 28 mm rings for repair of functional TR has demonstrated good immediate competency and satisfactory mid-term results [15], but only half of the studied population had severe FTR, and baseline RV function or annular size was not reported. However, others have reported residual rate of moderate or greater TR of up to 24% immediately after ring repair in patients with severe FTR and annular and RV dilation [16]. For patients with preoperative severe FTR, recurrence rates of ∼30% of moderate or greater TR at 1 year post ring annuloplasty have now been reported 2 large single-centre studies [17, 18]. Whether these suboptimal clinical results are related to progressive ventricular remodelling or untoward effects of aggressive annular reduction on RV performance is at this time unknown.

Our recent size and geometry analysis of 5 commercially available annuloplasty rings [19] revealed that size 28 mm rings have an area ranging from 462 to 538 mm^2^ with a mean area of 492 mm^2^. Three-dimensional echocardiography measured annular area in dilated and normal human right ventricles has been reported at 1566 and 1097 mm^2^ [20], respectively, and as such, annular reduction with this commonly used ring size would be associated with ∼68% area decrease of the annulus in a dilated right ventricle. Our prior experiments in sheep with normal hearts [7] and with RV remodelling and dysfunction implicate that such aggressive annular size reduction may have deleterious effects on RV strain patterns and performance. Furthermore, reanalysis of data from 2 large, randomized trials using undersized ring annuloplasty to treat functional mitral regurgitation found that severe annular reduction disproportionate to ventricular cavity size was the only independent predictor of recurrent mitral insufficiency potentially due to exacerbation of subvalvular tethering [21]. Clinical [22] findings support persistent leaflet tethering after mitral annuloplasty, a mechanism that may also potential come into play after undersized tricuspid annuloplasty. Therefore, aggressive tricuspid annular reduction may not only alter regional RV strains but also alter subvalvular geometry thus setting in motion a cycle of RV remodelling and dysfunction. As secondary TR and RV dysfunction are ‘synergistically related’ [23], these mechanisms may lead to progressive valve insufficiency.

Moderate annular reduction with prosthetic ring annuloplasty effectively treated FTR in our study but the degree of annular dilation achieved in our model was ∼35% versus normal healthy sheep of similar size [24]. In a pacing model of ovine biventricular failure with FTR and less pronounced annular dilation, suture annuloplasty of ∼50% reduced tricuspid insufficiency but with higher grade of residual TR [8]. These experimental findings are consistent with greater efficacy of ring versus suture annuloplasty reported in clinical studies [25, 26]. However, annular dilation in patients with RV dysfunction and dilation can be very pronounced and moderate annular reduction may not suffice to achieve a competent and durable repair. Therefore, additional leaflet and subvalvular procedures may be needed to avoid ‘corking’ the annulus with severe prosthetic undersizing. Leaflet extension [27] and approximation [28] and papillary muscle approximation [29] have recently been introduced to mitigate leaflet tethering and promote valve competence. Indeed, some authors advocate that in patients with severe FTR, annular dilation over 40 mm, and leaflet tethering, a concomitant procedure should supplement annular reduction. Such a multilevel approach to the tricuspid valvular complex may permit lesser degree of annular reduction while maintaining valvular competence and avoiding exacerbation of leaflet tethering and RV dysfunction that may be induced by aggressive downsizing. With detailed knowledge prosthetic ring area and geometry and improved imaging techniques to assess annular size and RV geometry, a more customized strategy to treating FTR may replace the ‘one size fits all’ approach currently more prevalent in surgical practice.

## CONCLUSION

Primary goal of valve repair for FTR it to achieve valvular competence, yet the fate of the right ventricle should not be forgotten. Therefore, it may be advantageous to maintain a balance between annular reduction and optimalization of RV geometry and function which may favour subsequent reverse remodelling. Our study demonstrates that in experimental ovine FTR, 50% annular reduction with a prosthetic ring was sufficient to abolish valvular insufficiency while maintaining regional RV function. Whether more aggressive prosthetic ring undersizing in both experimental and clinical setting disturbs this balance remains to be studied.

### Limitations

The result of the current study should be viewed in the context of several limitations. The data in the experiment were acquired in open-chest sheep under anaesthesia and direct extrapolation of these results into clinical practice should be done with caution. However, annular shape and geometry have been shown to be similar in awake and anaesthetized sheep [30]. Our model for functional TR was based on increased RV afterload due to pulmonary artery constriction which is distinctly different from clinical pulmonary hypertension arising from left-sided valvular lesions. We have previously used a rapid pacing biventricular failure model but feel that the current experimental preparation allows a more focused evaluation of tricuspid valve complex remodelling during the evolution of FTR. We assessed only epicardial strains using wired sonomicrometry crystals and no information regarding mid-wall and endocardial strains can be deemed from our study. Although the study demonstrated that moderate annular reduction did not alter RV free wall strain patterns, it is unclear whether further prosthetic undersizing would have a deleterious effect and this possibility requires further study. Experiment was performed as acute but under chronic condition, and our conclusions are limited to this setting. We have previously carried out a series of experiments investigating the influence of progressive downsizing of the tricuspid annulus with De Vega suture annuloplasty in sheep [8]. Using one size ring annuloplasty made this study clinically limited; however, future studies with higher range of ring size may be required to confirm our findings. Two CPBs are also very pertinent and are certainly a limitation of the study. However, both procedures (sonomicrometry implantation and ring annuloplasty) were performed with heart beating to minimalize the effect on myocardial function. Furthermore, each animal served as its own control to add power to the statistical conclusion of the study.

## Funding

This study was founded by internal grant from Meijer Heart and Vascular Institute at Spectrum Health.

**Conflict of interest:** Artur Iwasieczko and Marcin Malinowski were Peter C. and Pat Cook Research Fellows in Cardiothoracic surgery. No conflict of interest to declare.

## Data availability

Raw data were generated at Spectrum Health research office. All relevant data are within the manuscript and its supporting files. The data that support the findings of this study are available on request from the corresponding author.

## Author contributions

**Artur Iwasieczko:** Formal analysis; Investigation; Methodology; Supervision; Writing—original draft. **Monica Solarewicz:** Investigation. **Marcin Malinowski:** Formal analysis; Methodology. **Jared Bush:** Investigation; Methodology. **Brian MacDougall:** Investigation; Methodology. **Manuel Rausch:** Data curation; Formal analysis; Software; Validation. **Tomasz A. Timek:** Investigation; Project administration; Supervision; Writing—review & editing.

## Reviewer information

Interactive CardioVascular and Thoracic Surgery thanks Omar A. Jarral, Roman Gottardi and the other anonymous reviewer(s) for their contribution to the peer review process of this article.

## Notes

Presented at the 35th Annual Meeting of the European Association for Cardio-Thoracic Surgery, Barcelona, Spain, 13–16 October 2021.

## ABBREVIATIONS

FTR: Functional tricuspid regurgitation
RV: Right ventricular
TR: Tricuspid regurgitation
TRA: Tricuspid ring annuloplasty
